# Long-term results of the M^2^A-38-mm metal-on-metal articulation

**DOI:** 10.1186/s10195-018-0514-y

**Published:** 2018-12-07

**Authors:** Carlo Trevisan, Stefano Piscitello, Raymond Klumpp, Tonino Mascitti

**Affiliations:** UOC Ortopedia e Traumatologia, Ospedale Bolognini ASST Bergamo Est, Seriate, Italy

**Keywords:** Total hip arthroplasty, Metal on metal, Survivorship, Metal ions

## Abstract

**Background:**

Large-diameter head metal-on-metal (MoM) bearings in total hip arthroplasty (THA) are associated with increased whole blood levels of chromium (Cr) and cobalt (Co), adverse reactions to metal debris (ARMD) and poor survival rates. The prevalence of high metals concentrations, ARMD and the risk of revision surgery may vary significantly among different prostheses and long-term studies are few. This single-center study reports the long-term results of the 38-mm MoM bearing system.

**Materials and methods:**

Between 2003 and 2009, 80 patients received primary cementless THA using the large head metal-on-metal articulating surface M^2^A-38 cup (Biomet, Inc., Warsaw, IN, USA) at a single institution. Forty-five patients (53 hips) were retrospectively reviewed for a mean follow-up of 127 months.

**Results:**

Two cups were revised. The cumulative implant survival rate was 98% at 10 years and 74% at 13 years. In the whole sample, the median Co and Cr concentrations were 4.8 µg/L (IQR 1.2–4.9 µg/L) and 2.5 µg/L (IQR 0.6–3.0 µg/L), respectively. The incidence of Co or Cr levels > 7 μg/L was 15.5% and the incidence of ARMD was 3.8%. Co and Cr levels showed no correlation with cup inclination, Harris Hip Score, or total Hip Disability and Osteoarthritis Outcome score.

**Conclusions:**

Our results confirm that the problems of release of metal ions with the possible increase of metal circulating levels and of adverse reactions may also occur in the long term with this brand of MoM large head, and that a structured follow-up program is mandatory.

**Levels of evidence:**

Level 4.

## Introduction

Second-generation metal-on-metal (MoM) bearings in total hip arthroplasty (THA) were reintroduced in 1988 to reduce polyethylene particle-induced osteolysis and to increase stability using large-size femoral heads [1].

Medium-term outcomes seemed encouraging with a low rate of dislocation and wear and high patient satisfaction; this led to a worldwide increase in large-diameter MoM THA usage until a few years ago [2, 3].

Unfortunately, several reports have shown that large-diameter head MoM bearings can be responsible for adverse reactions to metal debris (ARMD) with the formation of peri-articular masses in some patients, referred to as pseudotumors [4], and higher than expected rates of revision [5].

Furthermore, MoM THA could lead to increased whole blood levels of chromium (Cr) and Cobalt (Co) with increasing concern about potential local and systemic toxic effects [6].

The prevalence of ARMD, the risk of revision surgery and blood ion concentrations may vary significantly among MoM prostheses depending on patient (female sex, young age, time since implantation), implant (design, head size, bilateral MoM) and surgical factors (acetabular component position, reduced contact patch to rim distance) [7–9].

Most of the studies on MoM were performed with medium-term follow-up (5–7 years) and some brands were preferably investigated.

Long-term follow-up of 38-mm MoM bearings are extremely scarce and could be useful to better define cost-effective surveillance strategies in similar implants.

This single-center study reports the long-term outcomes of 38-mm MoM bearing systems implanted between 2003 and 2009 in terms of implant survival, function, patient-reported outcome, radiological analysis and circulating metal ion levels.

## Materials and methods

Between 2003 and 2009, 80 patients received primary cementless THA using the large head metal-on-metal articulating surface M^2^A-38 cup (Biomet, Inc., Warsaw, IN, USA) at a single institution (Ospedale Bolognini Seriate).

Forty-five patients (53 hips) were retrospectively reviewed for a mean follow-up of 127 months (range 93–162). Twelve patients died and 13 were not contactable. Ten patients refused to present to the control for logistic or health problems; however, none of them were referred for revision of their implant.

There were 6 female and 39 male patients with a mean age of 70 ± 10 years at the last follow-up (range 42–86 years) and a mean body mass index of 27 ± 3 (range 18–36). The preoperative diagnoses included 44 cases of primary osteoarthritis and nine cases of avascular necrosis of the femoral head. The uncemented stems used were 29 PPF (Biomet, Inc.), 16 Bi-metric (Biomet, Inc.) and 8 CLS (Zimmer, Winterthur, Switzerland). Surgery was performed through a transgluteal (lateral) approach with the patient in the supine position under general or spinal anesthesia by the same senior surgeon (TM).

Clinical data were obtained at a follow-up visit. Patients were evaluated with the Harris Hip Score (HHS) [10] and the Hip Disability and Osteoarthritis Outcome Score (HOOS), Italian version LK 2.0 [11].

To quantify the percentage of patients with a successful result on HOOS, the patient-acceptable symptom state (PASS) estimated for HOOS pain and HOOS quality of life (QoL) was used as a cut-off point [12]. PASS is defined as the overall health state at which patients consider themselves to be feeling well: in the study of Maksymowych et al. this value resulted 91 for HOOS Pain and 83 for HOOS QoL [12]. Additionally, all patients were asked about possible cardiovascular or renal diseases and malignancies, other metal implants, smoking and drinking habits.

All patients were asked about intake of Co-containing or vitamin B12-containing nutritional supplements.

The Charnley classification was used to stratify patients into three categories in order to gain a better understanding of the variability in outcome [13, 14].

Anteroposterior and lateral radiographs of the hip and a pelvis overview radiograph were taken in the supine position at the follow-up visit. All radiographs except two were acquired with the same digital X-ray apparatus and evaluated by two of the authors (CT and SP).

The following parameters were determined—cup inclination and anteversion and stem inclination (analyzed with the digital planning software TraumaCad^®^; Brainlab, Inc.Westchester, IL USA), and radiolucent lines and osteolytic changes using Gruen zones and the De Lee Charnley zones, respectively [15, 16].

Femoral component fixation and stability were assessed by the Engh score [17].

Metal artifact reduction sequence (MARS) magnetic resonance imaging (MRI) was prescribed for patients following the management recommendations for patients with MoM hip replacement implants according to the MDA/2017/018 of the Medicine and Healthcare Products Regulatory Agency [18].

At the follow-up, each patient had a blood sample drawn through a non-metallic intravenous catheter into plastic tubes to eliminate contamination by contact with metal. Co and Cr were assayed in whole blood at the Industrial Hygiene and Toxicology Laboratory Spedali Civili di Brescia. Results were given in micrograms per liter; normal values were 0.05–1.0 μg/L for Co and 0.1–0.5 μg/L for Cr.

A three-group stratification scheme of ion levels in one or both metals was used: < 2 μg/L for the low-risk group, 2–7 μg/L for the medium-risk group and > 7 μg/L for the high-risk group [7, 18].

In order to better discriminate between the whole blood metal concentrations, patients were subdivided into three groups—those with monolateral MoMs, those with bilateral MoMs and those with monolateral MoMs and another hip or knee prosthesis.

Values are reported as median (interquartile range [IQR] and range), in compliance with current recommendations [19].

### Statistical analysis

Quantitative variables were described as mean, standard deviation and range for normal distributed variables and as median and IQR for non-normal variables. Qualitative variables were described as percentages.

Implant survival probabilities were computed using Kaplan–Meier analysis, counting revision of any component for any reason as the terminating event or at the end of the follow-up period [20]. The ANOVA test was used for comparison of normally distributed quantitative variables and the Kruskal–Wallis test was used for comparison of quantitative variables across the three implant groups when distribution was not normal.

Statistical analysis was performed using the STATA11 software package (Statistic Data Analysis, Statacorp, College Station, TX, USA). Values of *p* < 0.05 were considered significant.

## Results

At a mean follow-up of 127 months, two of the 53 hip were revised—the first for pain due to cup aseptic loosening after 84 months (no other data available) and the second for the presence of two pseudotumors and a high level of Co after 156 months.

**Fig. 1:**
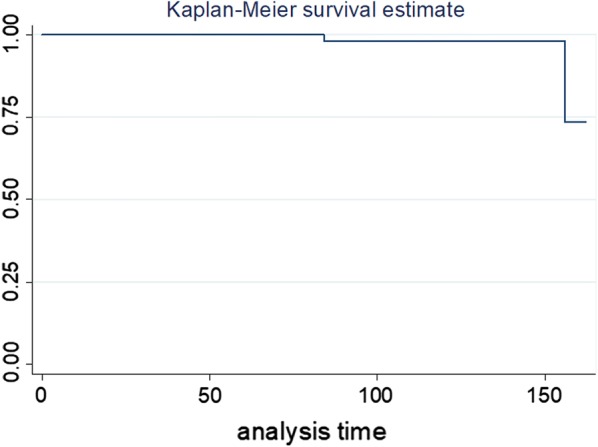
Kaplan–Meier analysis of implant survival probabilities, counting revision of any components for any reason as the terminating event or at the end of the follow-up period

The cumulative implant survival rate was 98% at 10 years but dropped to 74% at 13 years (Fig. 1).

Twenty-two patients were classified as Charnley Class A, 15 as Class B and 8 as Class C.

HHS and HOOS were not available for two patients and those of the two revised patients were not included in the analysis. The median HHS collected in 41 patients was 98 (IQR 95–100). The HHS of Class C was significantly lower than in Class A and B (Table 1).

**Table 1:** Harris Hip Score relative to Charnley Class

Charnley Class	Harris Hip Score, median (IQR)
A	99 (95–100)
B	98 (96–99)
C	91 (81–99)*

* *p* < 0.05 ANOVA test

Ninety-five percent of the patients were classified as excellent (85.7%) or good (9.5%) with the outcome of their operation and only two patients were classified as unfair (5%).

**Fig. 2:**
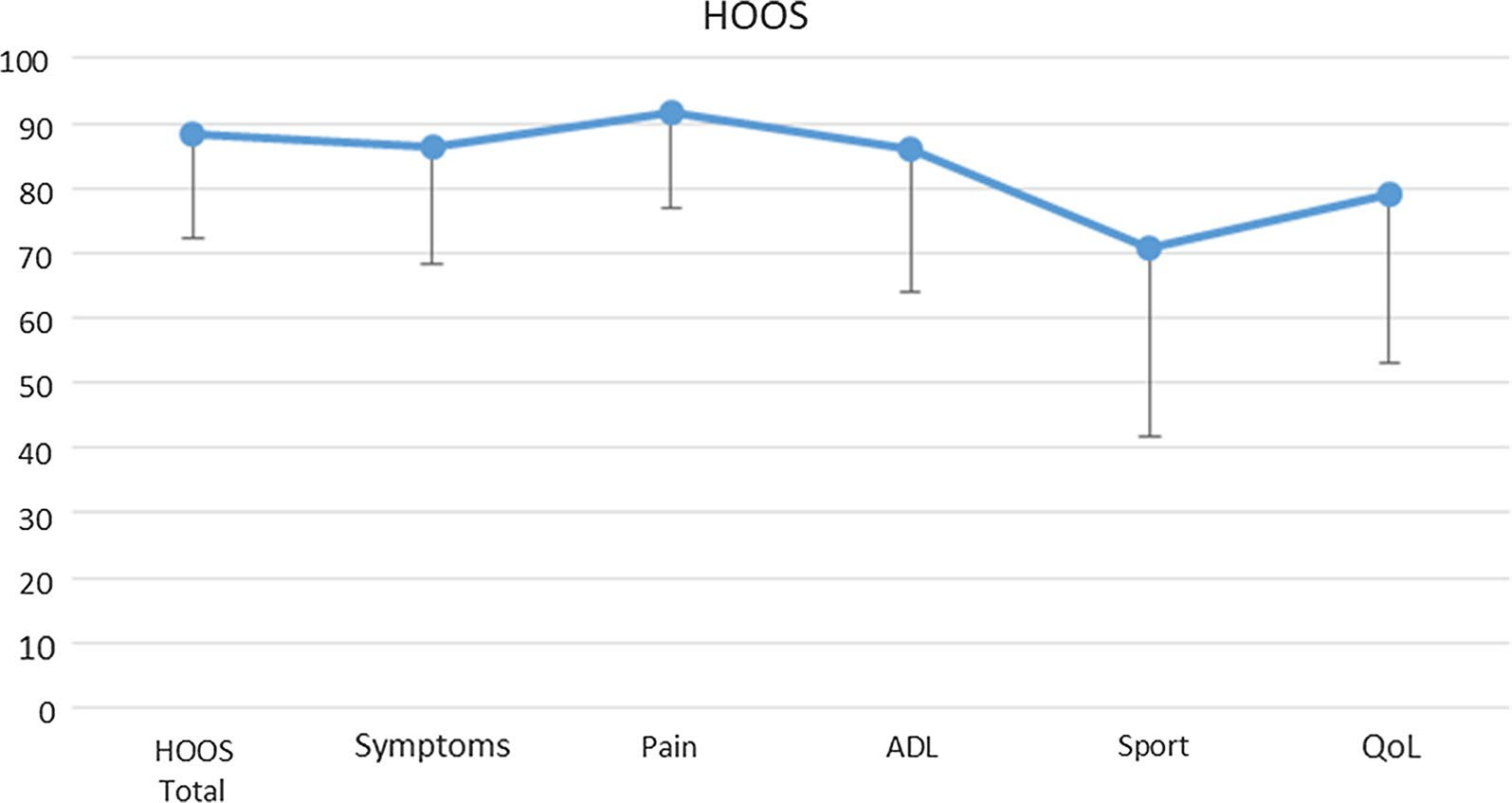
Hip disability and Osteoarthritis Outcome Score (HOOS) and subscale results

HOOS results are presented in Fig. 2. When PASS was used as the cut-off point, 33 of 42 patients (78.6%) for HOOS Pain and 27 of 42 patients (64.3%) for HOOS QoL had satisfactory results over a minimum period of 7 years after surgery.

No patients recalled cardiovascular, renal diseases or malignancies or other possible symptoms attributable to metal toxicity.

Forty-nine radiographs were available for assessment. The two revised cases were not included in the analysis and two radiographs acquired elsewhere were discarded for low quality.

Stem position showed an average inclination in varus of 1.7° (range 0–5). Average cup inclination was 40.1° (range 16-55) and average cup anteversion was 16.4° (range 0–29). Thirty-six of 49 cups (73.5%) were within the Lewinnek zone [21].

Zonal distribution of radiolucencies and lysis around implants is detailed in Table 2.

**Table 2:** Number of implants with radiolucencies or lysis around the stem or the cup

	Radiolucencies	Lysis
Stem–Gruen zones		
1	7	3
7	6	3
8	9	2
13	0	1
14	11	2
Cup–DeLee Charney zones		
1	1	5
2		7
3	1	2

Around the stem, radiolucent lines and osteolysis were predominantly present in the proximal femur (zones 1, 7, and 14). Around the acetabular component, radiolucent lines were scarce and osteolysis was mainly present in zones 1 and 2.

Twenty stems (40.8%) and 12 acetabular components (24.5%) presented at least one radiolucency or osteolysis; only 7 implants (14.3%) had both radiolucencies or osteolysis around the stem or the cup.

Assessment with the Engh score showed 32 osseointegrated stems (65.3%), 15 with suspected ingrowth (30.6%), two were suboptimum but stable (4.1%) and none were unstable.

No acetabular component was judged radiographically loose.

Seven patients underwent MARS MRI; two periacetabular pseudotumors were detected in the patient revised at 156 months. Three patients had a computed tomography scan to evaluate the dimensions of an osteolytic area in DeLee Charnley zone 2.

Whole blood metal analysis was performed in 42 patients; two patients refused and the patient revised at 84 months was not assessed.

In the whole sample, the median Co concentration was 4.8 µg/L (IQR 1.2–4.9 µg/L) and the median Cr level was 2.5 µg/L (IQR 0.6–3.0 µg/L).

Stratification of patients in the risk group is detailed in Table 3.

**Table 3:** Number of patients and percentage in each risk group for Co and Cr whole blood levels

Risk group	Co	Cr
Low (< 2 μg/L)	18 (42.8%)	27 (64%)
Intermediate (2–7 μg/L)	17 (40.5%)	12 (28.6%)
High (> 7 μg/L)	7 (16.7%)	3 (7.1%)

All patients with Co or Cr values > 2 µg/L had repeated their assessment after 3 months but none changed his/her stratification risk and the values looked stable.

Seven patients showed Co levels > 7 µg/L and three of them where those with Cr levels above the same threshold. One was the patient revised at 156 months. Three of them had a bilateral MoM. The six non-revised patients had stable components, two showing radiolucencies at the proximal femur and one an osteolytic area above the cup.

**Fig. 3:**
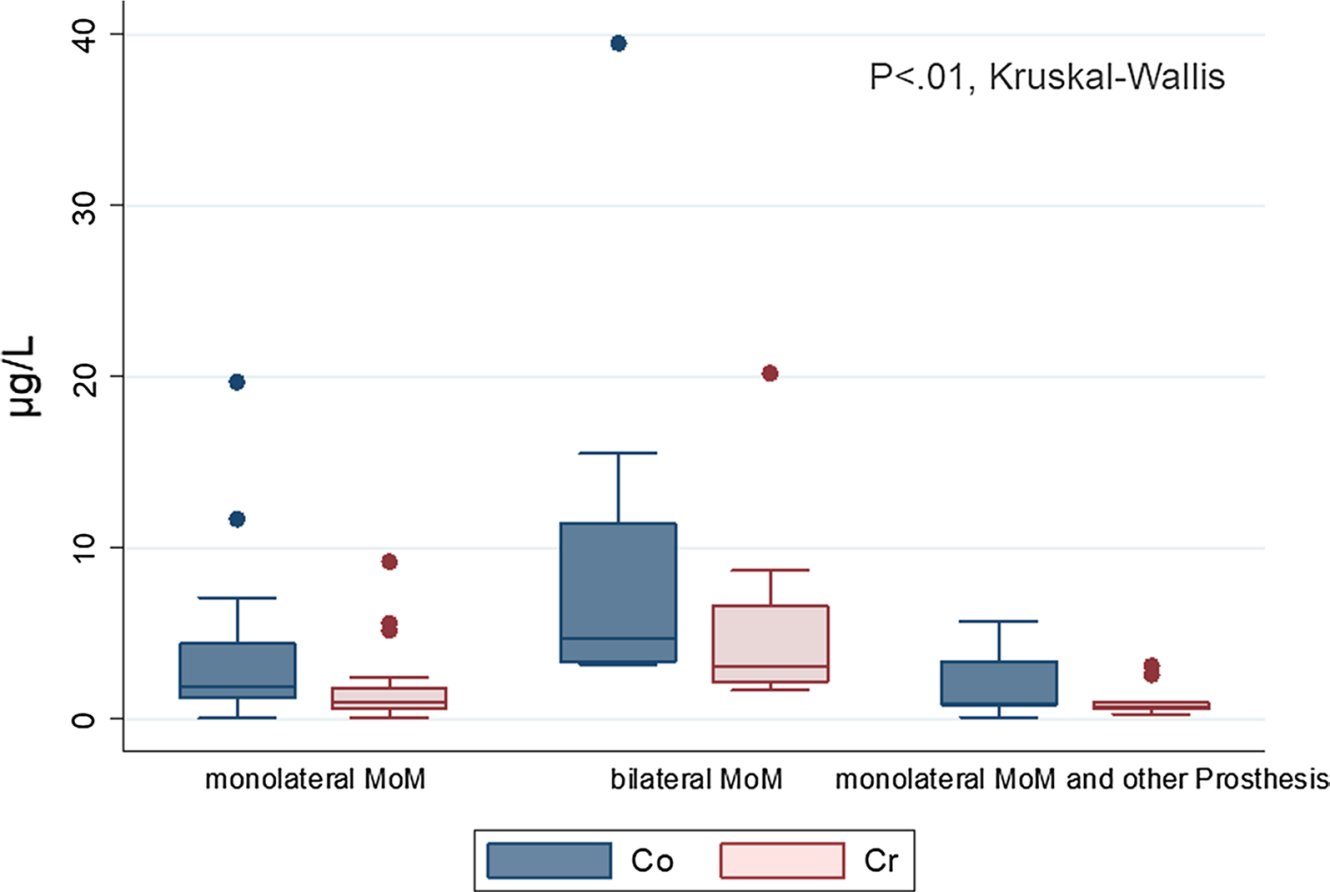
Box plots showing the whole blood levels of metal ions for cobalt (Co) and chromium (Cr) relative to the groups with monolateral MoM, bilateral MoM or monolaterale MoM with another hip or knee prosthesis. The boxes represent the median and interquartile range (IQR) and whiskers denote the range of data excluding outliers. Co and Cr levels were significantly higher in bilateral MoM (*p* < 0.01, Kruskal–Wallis)

Box plots for Co and Cr levels relative to the groups with monolateral MoMs, bilateral MoMs or monolaterale MoMs with another hip or knee prosthesis are shown in Fig. 3. Co and Cr levels were significantly higher in bilateral MoMs (*p* < 0.01, Kruskal–Wallis).

Co and Cr levels showed no correlation with cup inclination, cup anteversion, HHS, total HOOS and HOOS subscales.

## Discussion

There is limited knowledge about the long-term results (> 10 years) of MoM THA with a 38-mm head regarding implant survival, function, patient-reported outcome, radiological analysis and circulating metal ion levels. In a literature revision on MoM hip arthroplasty by Jantzen et al. [6], only three of 43 studies had a follow-up of > 100 months; two dealt with small-head MoMs and a third dealt with hip resurfacing arthroplasty.

Registry data report a disappointing survival rate ranging between 56.7% and 88.9% at 10 years for large-head MoM THA of different diameters and brands [7].

In our study of 53 THAs with 38-mm MoM articulation of a single brand, the cumulative survival rate was very good at 10 years (98%) and dropped to 74% at 13 years after the follow-up program. Two cup revisions were recorded at 84 and 156 months. The cup revision at 84 months was performed elsewhere and was due to aseptic loosening and pain, and no data were available about local reaction on metal debris. The second patient revised at 156 months underwent surgery as a result of follow-up for the presence of two para-acetabular pseudotumors and high levels of circulating Co, even though both components were stable. In this case, the level of circulating Co was the main reason for performing MARS MRI since the standard radiographs showed only some radiolucent lines in the proximal femur and the patient was completely asymptomatic.

Our results are in line with medium- and long-term survival for MoM THA with heads of a similar size reported for the Pinnacle system by various authors.

Matharu et al. reported 39 revisions in 578 MoM THAs at a mean time of 3.5 years with a cumulative 8-year survival rate of 88.9% and with 44% of the revisions performed for ARMD [22]. In the study by Lainiala et al., the application of a new surveillance protocol revealed 23 new cases of ARMD and the 9-year survival of this cohort declined from 96 to 86% [23].

Langton et al. revised 71 of 489 MoM Pinnacle hips with a survival rate of 83.6% for the whole cohort at 9 years. All but one of the revisions were carried out for ARMD, with the majority of explanted devices exhibiting signs of taper junction failure and a significant number of devices manufactured out of their specifications [24].

In a retrospective study by Atrey et al. on a consecutive series of 469 hips, 29 were revised (12 for ARMD) with a mean survivorship of the implant of 92.8% at a median time to follow-up of 84 months [25]. Umar et al. reported the results of a cohort of patients aged ≤ 55 years; they performed 12 revisions in 109 hips (7 for ARMD) with an implant survival rate of 88.1% at a mean age of 10 years [26].

Seven of our patients showed Co levels > 7 μg/L, which means a 15.5% incidence of high levels of Co or Cr. In the worst scenario, the occurrence of adverse reactions to metal debris observed after the application of the MHRA recommendations for imaging had an incidence of 3.8%, if we also suppose that the patient revised at 84 months had an ARMD.

In our cohort, blood metal levels, percentage of patients above the MHRA safety threshold and the incidence of ARMD are similar to those reported for 36-mm Pinnacle heads in different studies (Table 4).

**Table 4:** Comparison of median blood metal levels and percentage of patients with high metal levels and ARMS in studies with MoM bearings of similar head size (36–38 mm)

	Blood metal levels median (μg/L)			% patients with Co or Cr levels > 7μg/L	% patients with ARMD
Matharu et al. [22]	WB	Co 2.06 (0.83–3.71)^a^	Cr 1.25 (0.83–2.03)^a^	8.7	2.9
Lainala et al. [23]	WB mono	Co 1.5 (0.7–4.0)^a^	Cr 1.1 (0.7–1.7)^a^	13.9	9.8
	WB bil	Co 5.0 (1.6–8.9)^a^	Cr 1.9 (1.1–3.3)^a^		
Langton et al. [24]	WB mono	Co 3.62 (0.86–19.7)^b^	Cr 3.62 (0.88–26.2)^b^	–	19.9
	WB bil	Co 9.54 (1.42–27.0)^b^	Cr 9.72 (0.47–26.3)^b^		
Atrey et al. [25]	WB	Co 2.9 (0.2–82.3)^b^	Cr 2.1 (0.2–35.3)^b^	21	2.6
Umar et al. [26]	S	Co 4.3	Cr 6.8	18	3
This study	WB mono	Co 1.9 (0.1–19.7)^b^	Cr 1.0 (0.1–9.2)^b^	16.7	3.8
	WB bil	Co 4.7 (3.2–39.5)^b^	Cr 3.1 (1.7–20.2)^b^		

*WB* whole blood, *S* serum; *mono* monolateral MoM, *bil* bilateral MoM

^a^Interquartile range

^b^Range

As observed in a study by Atrey et al. [25], the Cr levels were never independently raised above Co, as in our patient. Furthermore, it has been suggested that Co (rather than Cr) is the more active ion that initiates an inflammatory reaction and it has a trend towards larger blood concentrations with longer follow-up [24, 27].

These observations and our results add to the argument that Cr assessment may not be necessary [28].

Overall, 95% of the non-revised patients showed excellent or good function > 10 years after implantation and standard radiographs showed no evidence of implant mobilization. Most of these patients were happy with their hip replacement as they were able to perform activities of daily living and work without compromise.

Nevertheless, even this particular brand of MoM bearing seems to have a similar incidence of the problems of elevation of the circulating levels of metals and ARMD that have characterized the other brands which have been more thoroughly investigated.

The limitation of this study is the significant number of patients who were lost-to-follow-up. We were not able to collect any reliable information about the outcomes of the implants of these patients but the lost patients may make our results too optimistic. Another limitation concerns pelvis radiographs taken in the supine position; this may cause a slight underestimation of 4° to 6° of cup inclination and anteversion [29].

In conclusion, as far as we know, this is the only study with a follow-up of > 10 years involving a hip prosthesis using an MoM coupling with a 38-mm head that does not concern the Pinnacle system.

Our results confirm that the problems of release of metal ions with a possible increase of metal circulating levels and of adverse reactions may occur with a very similar probability to that observed for the most investigated systems.

Therefore, a structured systematic follow-up program according to the MHRA guidelines is appropriate for any large-head MoM implant.
